# Contribution of p53-dependent and -independent mechanisms to upregulation of p21 in Fanconi anemia

**DOI:** 10.1371/journal.pgen.1011474

**Published:** 2024-11-07

**Authors:** Xavier Renaudin, Baraah Al Ahmad Nachar, Benedetta Mancini, Anna Gueiderikh, Noémie Louis-Joseph, Frédérique Maczkowiak-Chartois, Filippo Rosselli

**Affiliations:** 1 CNRS UMR9019, Université Paris-Saclay, Gustave Roussy Institute Cancer Campus, Villejuif, France; 2 Equipe Labellisée Ligue Nationale Contre le Cancer, Villejuif, France; St Vincent’s Institute, AUSTRALIA

## Abstract

Abnormal expression of the cell cycle inhibitor and p53 target *CDKN1A*/p21 has been associated with paradoxical outcomes, such as hyperproliferation in p53-deficient cancer cells or hypoproliferation that affects hematopoietic stem cell behavior, leading to bone marrow failure (BMF). Notably, p21 is known to be overexpressed in Fanconi anemia (FA), which is a rare syndrome that predisposes patients to BMF and cancer. However, why p21 is overexpressed in FA and how it contributes to the FA phenotype(s) are still poorly understood. Here, we revealed that while the upregulation of p21 is largely dependent on p53, it also depends on the transcription factor microphthalmia (MITF) as well as on its interaction with the nucleolar protein NPM1. Upregulation of p21 expression in FA cells leads to p21 accumulation in the chromatin fraction, p21 immunoprecipitation with PCNA, S-phase lengthening and genetic instability. p21 depletion in FA cells rescues the S-phase abnormalities and reduces their genetic instability. In addition, we observed that reactive oxygen species (ROS) accumulation, another key feature of FA cells, is required to trigger an increase in PCNA/chromatin-associated p21 and to impact replication progression. Therefore, we propose a mechanism by which p21 and ROS cooperate to induce replication abnormalities that fuel genetic instability.

## Author summary

Fanconi anemia (FA) is a rare genetic disorder characterized by bone marrow failure and a predisposition to cancer caused by biallelic mutations in genes encoding proteins involved in the FANC/BRCA DNA repair pathway. Bone marrow failure has been attributed to the abnormal overexpression of the p53/p21 axis in response to the inherent DNA damage accompanying the loss of the FANC/BRCA pathway, leading to cell cycle arrest and subsequent hematopoietic stem cell exhaustion. Here, we revealed an alternative pathway that contributes to p21 overexpression independent of p53. Indeed, we found that in *TP53*-null cells, the loss of the FANC pathway still leads to p21 overexpression. We demonstrated that this overexpression is dependent on the transcription factor MITF, which increases *CDKN1A* mRNA (which encodes p21), and on the NPM1 protein, which directly stabilizes p21. Both mechanisms are independent of DNA damage and/or DNA damage signaling, and therefore, this study provides a shift in the paradigm explaining the bone marrow failure observed in Fanconi anemia. Notably, we demonstrated that, in association with the known higher level of ROS in FA cells, the overexpression of p21 contributes to generating replication stress, which contributes to their genetic instability.

## Introduction

The primary function of the FANConi/BReast CAncer (FANC/BRCA) tumor suppressor pathway is to ensure accurate chromosome duplication and segregation, a vital process in preventing bone marrow failure (BMF), cancer susceptibility, and the chromosomal fragility syndrome Fanconi anemia (FA) [1–5]. Integrated within the DNA damage response (DDR) network, this pathway orchestrates a set of highly regulated processes, ensuring faithful DNA repair and replication in coordination with cell cycle progression while maintaining genetic stability. Over 20 proteins associated with the FANC/BRCA pathway have been identified, including regulators such as FANCM, the FANCcore complex (FANCA, FANCG, FAAP20, FANCC, FANCE, FANCF, FANCB, FANCL, FAAP100) and the FANCD2/FANCI heterodimer and effectors involved in processes such as homologous recombination, nucleotide excision repair, and G-quadruplex resolution, such as BRCA1/FANCS, BRCA2/FANCD1, PALB2/FANCN, RAD51/FANCR, XPF/ERCC4/FANCQ and BRIP1/BACH1/FANCJ [6].

A key biochemical event within the FANC/BRCA pathway is the monoubiquitination of FANCD2 and FANCI mediated by the FANC core complex [7,8]. This occurs during DNA replication in response to delayed or stalled replication forks, facilitating their rescue. Apart from DDR abnormalities, cells lacking functional FANC/BRCA pathways exhibit a proinflammatory phenotype characterized by high intracellular levels of reactive oxygen species (ROS) and constitutive activation of stress signaling pathways [9–18]. These cells exhibit imbalances in ribosome biogenesis associated with altered nucleolar homeostasis and a reduced translation rate [19,20] but also overactivation of the p53-p21 axis, a key determinant of cell cycle progression and proliferation [21–23]. p53-p21 overactivation has been demonstrated to affect hematopoietic stem and progenitor cells (HSPCs) in FA patients and mice, contributing to BMF development [22]. However, the origins and contributions of these abnormalities to the FA phenotype(s) remain poorly understood.

Our findings revealed that in FA cells several pathways/proteins contribute to p21 overexpression beyond the key role of the upregulated p53. Indeed, p21 overexpression in FANC pathway-deficient cell is the result of the convergence of transcription-dependent and transcription-independent events associated with a) DNA damage (ATM-p53 axis), b) nucleolar abnormalities (NPM1), and c) unregulated activation of cellular stress signaling pathways that, in turn, activate the transcription factor microphthalmia (MITF) [18]. Surprisingly, we observed that p21 overexpression and intracellular ROS in FA cells collectively led to changes in replication characterized by reduced single-cell EdU incorporation. This replicative stress contributes to the lengthening of S phase, hypoproliferative status, and genetic instability, which are major determinants of BMF in this syndrome.

## Results

### Loss of function of the FANC pathway increases p21 expression in both p53-dependent and p53-independent manners

The p21 protein, encoded by the *CDKN1A* gene, is a major p53 target [24], whose transcriptional activity is, in turn, induced by the DDR apex kinases ATM and ATR in response to genotoxic stress [25,26]. Additionally, p53 can be activated in response to nucleolar/ribosomal stress in an ATM/ATR- and phosphorylation-independent manner [27,28]. Both stress conditions have been described in FA, prompting us to investigate the origins of p53-p21 overexpression in FANC pathway-deficient cells.

**Fig 1:**
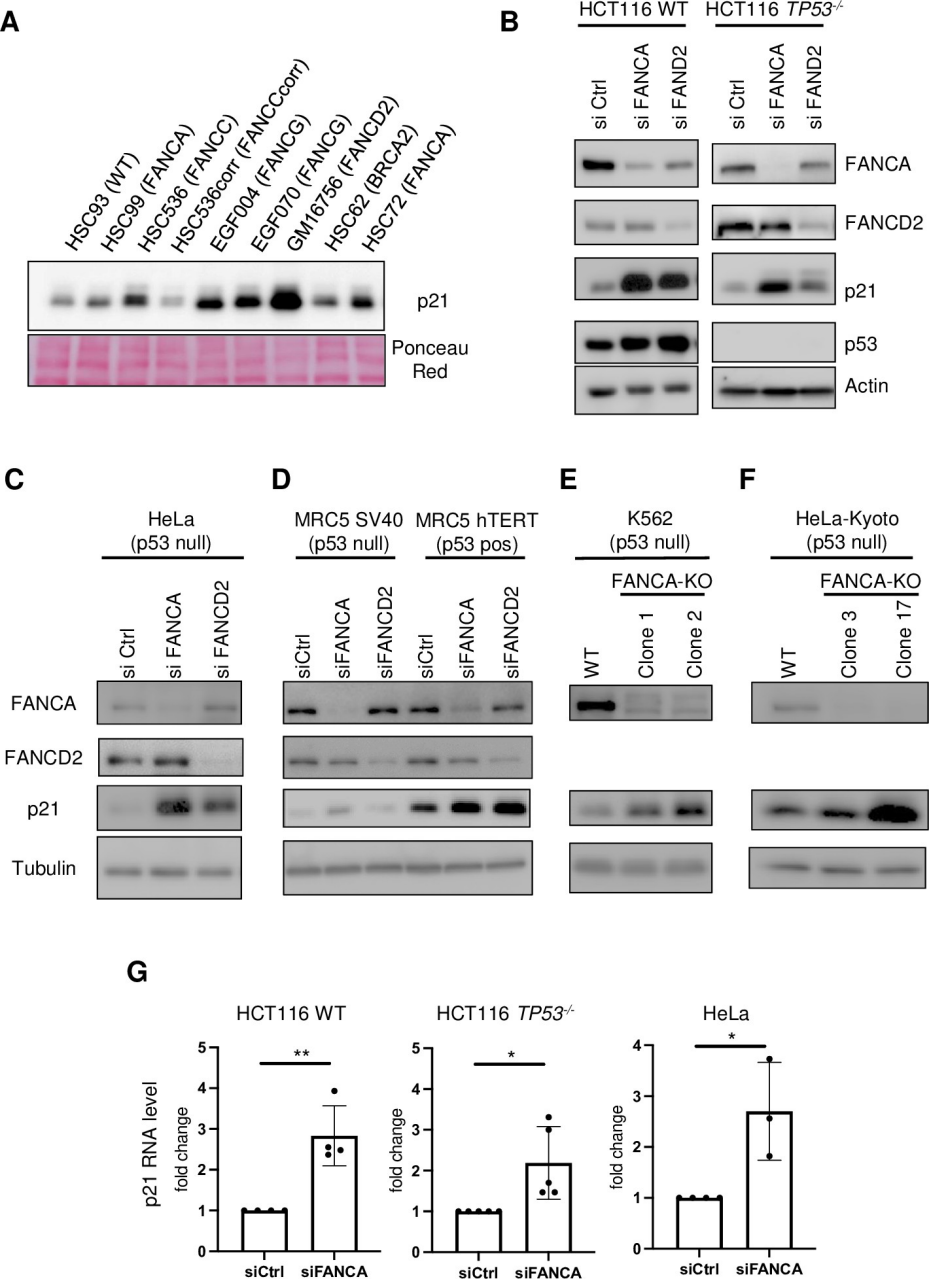
*CDKN1A*/p21 overexpression in FANCA-deficient cells is a p53-dependent and p53-independent event. (A-F) Representative Western blots showing the levels of p21 and the indicated proteins in (A) WT, FANC-deficient or FANC-corrected lymphoblasts. Ponceau Red staining of the membrane served as a loading control. (B) HCT116 WT or HCT116 *TP53*^-/-^ cells were transfected with siRNA control (siCtrl) or siRNA targeting *FANCA* (siFANCA) or *FANCD2* (siFANCD2). Actin was used as a loading control. (C) HeLa cells and (D) MRC5 SV40 and MRC5-hTERT cells transfected with siRNA control (siCtrl) or siRNA targeting *FANCA* (siFANCA) or *FANCD2* (siFANCD2). Tubulin was used as a loading control. (E) K562 parental cells and two FANCA KO clones and (F) HeLa Kyoto parental cells and two FANCA KO clones. In C to F, tubulin was used as a loading control. (G) Histograms showing normalized mRNA levels of p21 (*CDKN1A*) measured by qPCR in HCT116 WT, HCT116 *TP53*^-/-^ or HeLa cells transfected with siRNA control (siCtrl) or siRNA targeting *FANCA* (siFANCA) for 72 h. Each point represents an individual experiment. * p<0.05; ** p<0.01.

The p21 overexpression was observed following the siRNA-mediated depletion of FANCA or FANCD2 and was normalized by the ectopic expression of the corresponding WT gene in human lymphoblasts (FANCCcorr, **Fig 1A**; FANCAcorr, **S1B Fig**). This finding emphasizes that upregulation of the expression of p21 is a direct consequence of the loss of function of an FA-associated protein and is not secondary to additional genetic alterations due to the absence of a functional DDR pathway.

Consistent with previous studies, compared with FANC pathway-proficient cells, patient-derived EBV-immortalized lymphoblasts deficient in the FANC core complex (FANCA, FANCC, or FANCG), FANCD2, or FANCD1/BRCA2 exhibited elevated p21 expression **(Fig 1A)**. Accordingly, siRNA-mediated depletion of FANCA or FANCD2 increased the expression of p53 and p21 in HCT116 cells **(Fig 1B, left panel)**. However, unexpectedly, p21 overexpression was also observed, although at an obviously lower level **(Fig 1D)**, in p53-deficient HCT116, HeLa and MRC5-SV40 cells in which FANCA or FANCD2 expression was transiently downregulated by siRNA transfection as well as in K562 cells in which *FANCA* was knocked out (KO) via the CRISPR/Cas9 approach **(Fig 1B–1E).** We also generated a new HeLa Kyoto *FANCA*-KO cell line that does not monoubiquitinate FANCD2 **(S1A Fig)** and found it overexpresses p21 **(Fig 1F).** Moreover, to further demonstrate that in FANCA-deficient cells, p21 overexpression is partially independent of p53, we treated the cells with the pifithrin-α, a known inhibitor of p53. While this inhibitor downregulates p21 in WT cells (HSC72corr), we observed that the p21-overexpression in FANCA^-/-^ lymphoblasts is resistant to pifithrin-α (PFT-α) **(S1B Fig)** suggesting other mechanisms may be involved to sustain p21 overexpression.

Subsequent flow cytometry analysis of cell populations stained with propidium iodide (PI, a DNA marker) and an anti-p21 antibody demonstrated that p21 is overexpressed in all cell cycle phases **(S1C Fig)** independent of the p53 status of the cells. Moreover, in FANCA-deficient cells, p21 overexpression was associated with increased transcription of its mRNA, as determined by qRT–PCR **(Fig 1G)**, suggesting that transcription factors (TFs) other than p53 mediate *CDKN1A* expression in FA cells.

Thus, our findings indicate that in FA, p21 overexpression is predominantly caused by p53, but that in its absence, other pathways can still support p21 upregulation in response to FA deficiency.

### The DDR partially contributes to p21 overexpression in FANC pathway-deficient cells

**Fig 2:**
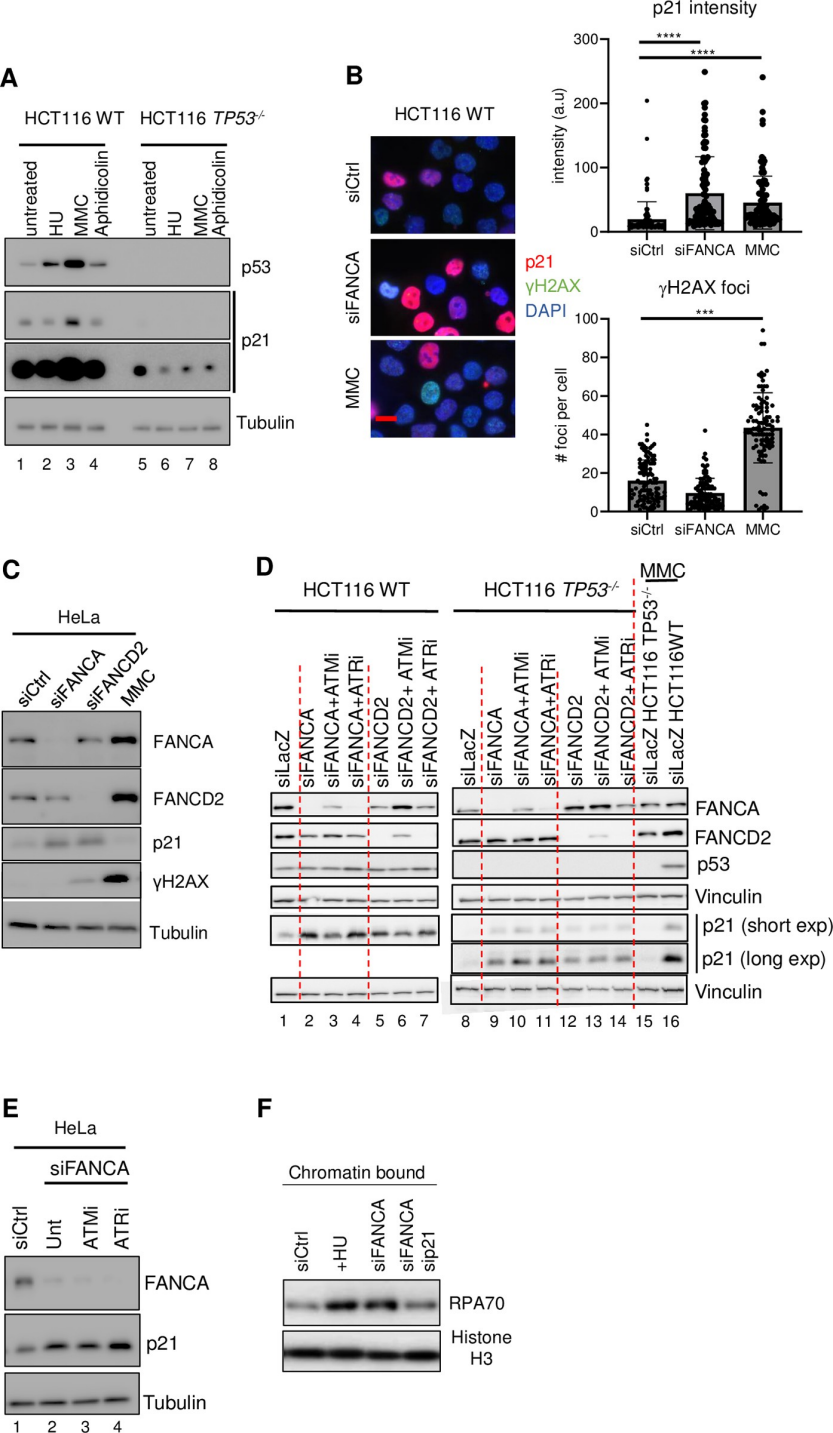
CDKN1A/p21 overexpression in FANCA-deficient cells is partially dependent on DNA damage-ATM-p53 signaling. (A) Representative Western blot showing p53 and p21 expression in response to DNA damaging agents in WT or TP53^-/-^ HCT116 cells. The cells were treated overnight with 1 mM HU, 200 ng/mL MMC or 0.5 μM Aph. Tubulin served as a loading control. (B) Immunofluorescence analysis of the level of p21 and the number of γH2AX foci in HCT116 WT cells transfected with either untargeted (siCtrl) siRNA or siRNA targeting *FANCA* (siFANCA) and treated with or without 200 ng/mL MMC overnight. The scale bar is 20 μm. Quantification of n = 3 experiments with at least 50 cells per experiment. (C-E) Representative Western blots showing the expression levels of the indicated proteins in (C) HeLa cells 72 hours after siRNA-mediated depletion of FANCA or FANCD2 or following overnight exposure to 200 ng/ml MMC. Tubulin served as a loading control. (D) FANCA- or FANCD2-depleted WT or TP53^-/-^ HCT116 cells were analyzed 72 h after transfection or treatment with MMC as described above. ATMi (10 μM) and ATRi (100 nM) were added for 18 h. Vinculin served as a loading control. Red dotted lines separate the different siRNA conditions. (E) FANCA-depleted HeLa cells. The cells were transfected for 72 h, and ATMi (10 μM) or ATRi (100 nM) was added during the last 18 h. (F) Representative Western blot showing the amount of chromatin-bound RPA70 protein in HeLa cells treated with HU (dose) or following siRNA-mediated depletion of FANCA alone or FANCA and p21. H3 was used as a loading control.

Next, we sought to determine the contribution of the DNA damage/replication stress signaling-p53 axis to the p21 overexpression observed in FA cells. We first challenged p53-proficient and p53-deficient HCT116 cells with mitomycin C (MMC), aphidicolin (APH) or hydroxyurea (HU). The anticancer drug MMC induces bulky DNA monoadducts, interstrand cross-links (ICLs) and oxidative lesions, targeting DNA in all phases of the cell cycle. By inhibiting the activity of replicative DNA polymerases, APH and HU generate replication stress by delaying/blocking replication fork progression, challenging exclusively S-phase cells. All of the tested genotoxins increased p53 expression **(Fig 2A)**. However, consistent with the key role of the p53-p21 axis in mediating G1 checkpoint activation to counteract the S-phase entry of damaged cells, p21 was significantly induced only in response to MMC **(**Lane 3 in **Fig 2A)**. Importantly, in p53-deficient cells, MMC exposure did not increase p21 expression **(Fig 2A** (Line 7)**, 2C and 2D** (Line 15)**)**, confirming the pivotal role of p53 in DNA damage-induced p21 expression in G1.

In FANCA-proficient cells, MMC exposure was associated with an increased H2AX phosphorylation (γH2AX), which is indicative of DNA damage (mainly DNA double-strand breaks) and ATM/ATR activation **(Fig 2B and 2C)**. The MMC-induced increase in γH2AX signals observed in p53-deficient HeLa cells suggested that ATM/ATR signaling was intact in these cells **(Fig 2C)**. Notably, independent of their p53 status, we detected only a marginal increase, if any, in γH2AX following FANCA or FANCD2 depletion **(Fig 2B and 2C)**. Moreover, irrespective of their p53 status, only a marginal decrease in p21 overexpression was observed in FANCA- or FANCD2-depleted cells after ATM inhibition **(**compare lanes 2 and 3, and 5 and 6 in **Fig 2D**; and compare lanes 2 and 3 in **Fig 2E)**. These data suggest that loss of FANCA or FANCD2 function has limited, if any, consequences on the level of endogenous DNA damage and ATM activation. However, consistent with previously published data showing no phosphorylation of CHK1, the main target of ATR in FA-deficient cells [29], ATR inhibition did not affect p21 expression **(**compare lanes 2 and 4, and 5 and 7 in **Fig 2D**; and compare lanes 2 and 4 in **Fig 2E)**.

Subsequently, using the chromatin accumulation of RPA70 as a readout, we investigated whether FANCA-deficient cells experience endogenous replication stress. Cells treated with HU served as a positive control. Depletion of FANCA resulted in clear chromatin accumulation of RPA70 **(Fig 2F)**, suggesting that FA cells undergo endogenous replication stress. We revealed that the chromatin accumulation of RPA in FANCA-deficient cells appear to be partially dependent of p21 overexpression in absence of p53 **(Fig 2F)**.

Taken together, the above observations indicate that in untreated FANC pathway-deficient cells, other events than DDR signaling contribute to p21 overexpression.

### MITF participates in the transcriptional induction of p21 in FANCA-deficient cells

**Fig 3:**
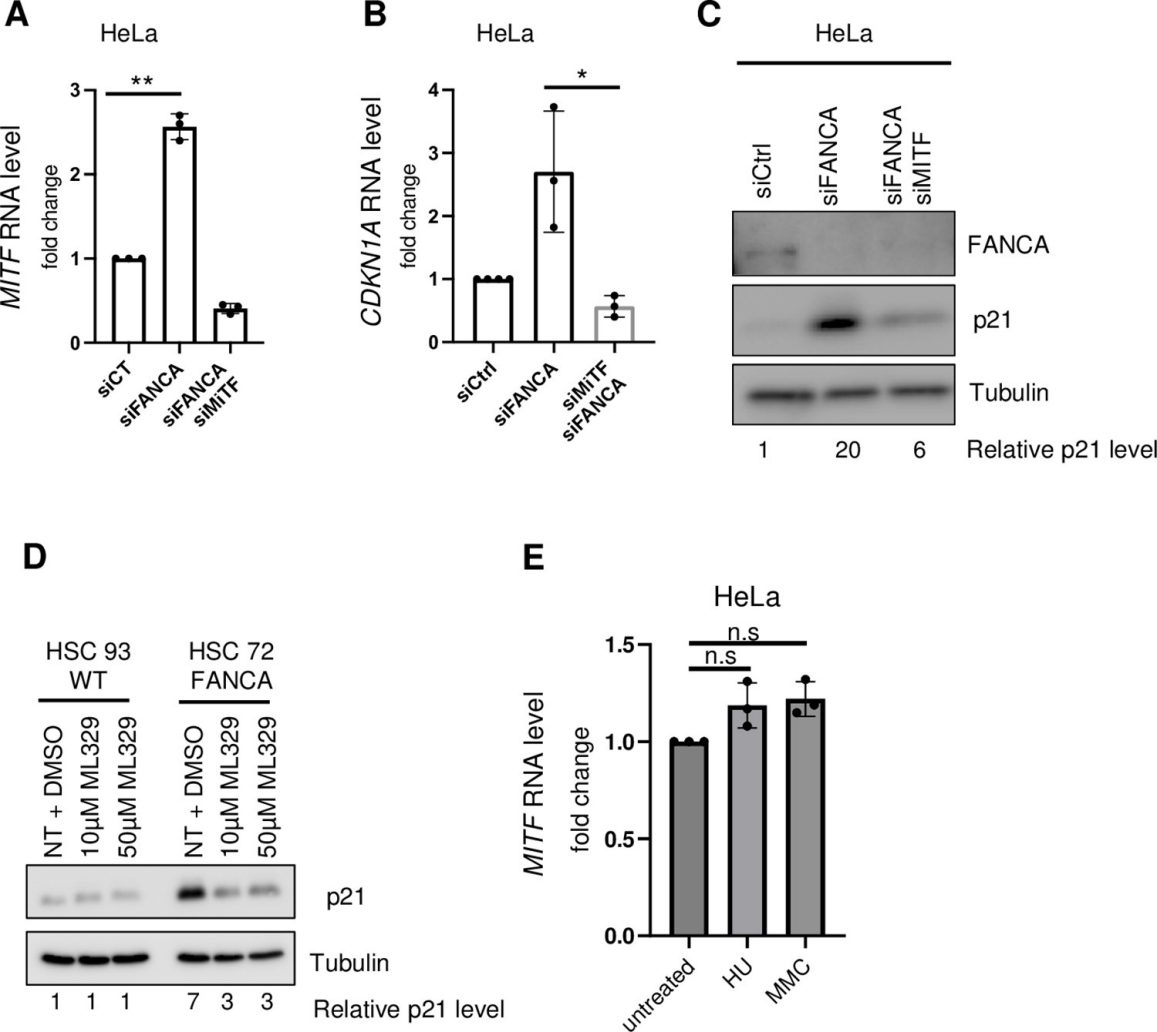
MITF is a TF that controls the level of *CDKN1A*/p21 in cells deficient in p53 and FANCA. (A and B). Histograms showing normalized mRNA levels for *MITF* (A) and *CDKN1A* (B) measured by qPCR in HeLa cells transfected with siRNA control (siCtrl), siRNA targeting *FANCA* (siFANCA) or siRNA targeting *FANCA* and *MITF* (siFANCA siMITF) for 72 h. Each point represents an individual experiment. * p<0.05; ** p<0.01. n = 3. (C) Western blot showing the level of p21 expression in HeLa cells 72 hours after siRNA-mediated depletion of FANCA or FANCA and MITF. Tubulin served as a loading control. (D) Western blot showing the level of p21 expression in WT (HSC93) or FANCA-deficient (HSC72) lymphoblasts 18 hours after exposure to the MITF inhibitor ML329. Tubulin served as a loading control. (E) Histograms showing normalized mRNA levels of *MITF* measured by qPCR in HeLa cells after treatment with HU (1 mM) or MMC (200 ng/mL) for 18 h.

Having observed **(Fig 1G)** that *CDKN1A* expression is increased in p53-deficient cells, we investigated which TFs other than p53 might cooperate to increase its expression. This led us to examine the potential role of the TF microphthalmia (MITF), which is known to induce *CDKN1A* expression through its binding to the E-box motif CATGTG in its promoter [30]. Indeed, previous studies have shown that MITF is overexpressed in FANC pathway-deficient cells and that it is involved in BMF in FANCA-deficient mice in an as yet undefined manner [18,31,32]. Here, we demonstrated that FANCA depletion in HeLa cells was associated with the induction of *MITF*, *CDKN1A* and p21 **(Fig 3A to 3C)**. MITF downregulation in FANCA-depleted HeLa cells strongly reduced *CDKN1A* expression and p21 protein levels **(Fig 3A–3C)**. In addition, after treating FANCA-deficient p53-proficient lymphoblasts with the MITF inhibitor ML329 [33], we again observed a strong reduction in the intracellular level of p21 **(Fig 3D)**. Thus, our data demonstrate that in FANCA-deficient cells, MITF contributes to the abnormal increase in *CDKN1A*/p21 expression. Moreover, DNA damage and replication stress did not lead to MITF induction **(Fig 3E)**, confirming that the level of endogenous DNA damage is not sufficient to trigger this axis of response.

However, even in p53-deficient and MITF-depleted/inhibited FANCA-deficient cells, p21 protein expression remained above the basal level observed in FANCA-proficient cells **(Fig 3C and 3D)**. Thus, our observations suggest that additional events in cooperation with p53 and MITF increase p21 expression downstream of FANCA deficiency.

### Nucleolar/ribosomal stress cooperates with p21 overexpression in FANC pathway-deficient cells

**Fig 4:**
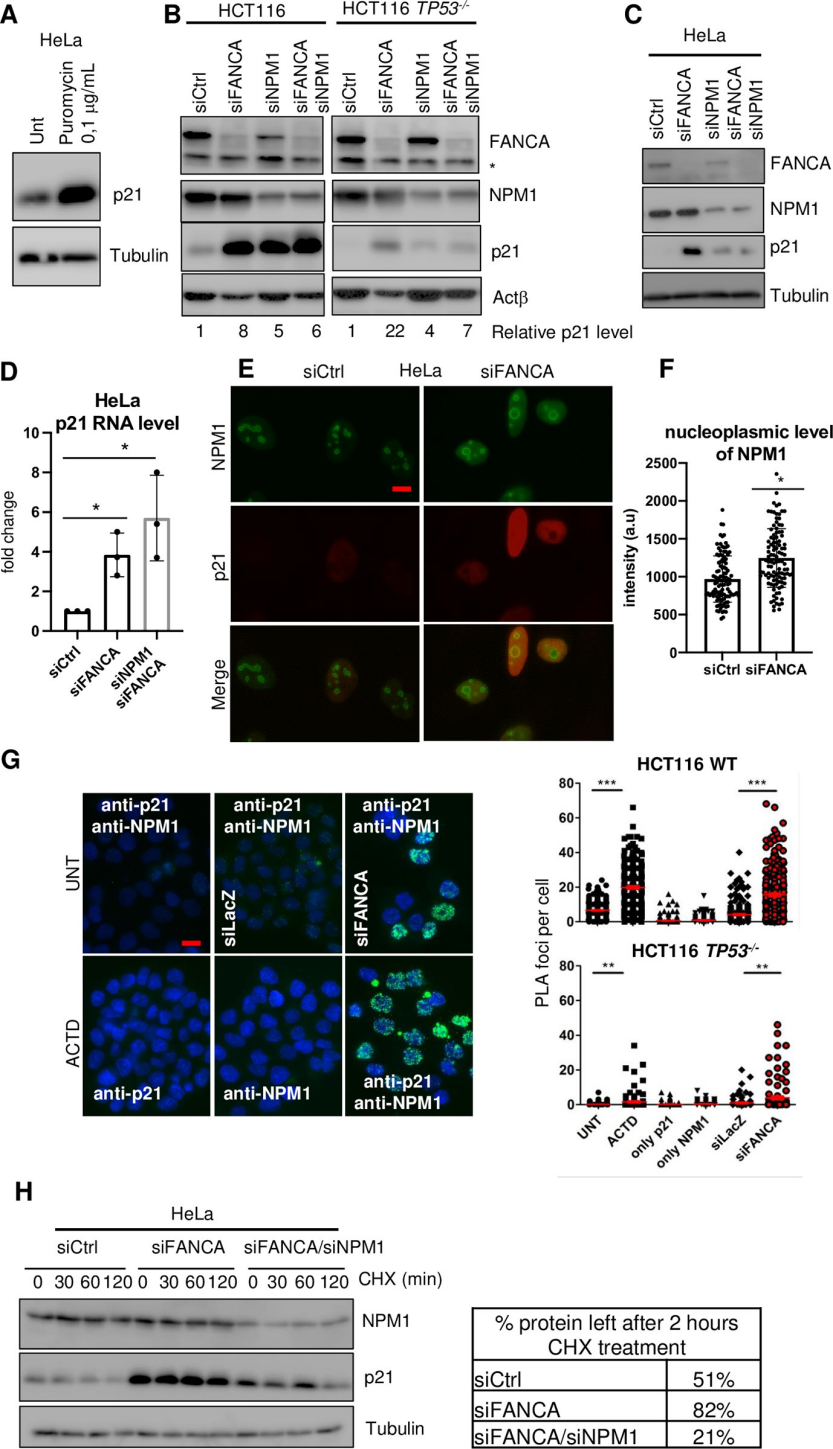
Nucleolar stress contributes to p21 stabilization in FANCA cells. (A-C) Western blot showing the levels of the indicated proteins in (A) HeLa cells after treatment with 0.1 μg/mL puromycin for 24 h. Tubulin served as a loading control. (B) HCT116 WT and HCT116 *TP53*^*-/-*^ cells were transfected with siRNA control (siCtrl), siRNA targeting FANCA (siFANCA), siRNA targeting NPM1 (siNPM1) or siRNA targeting both NPM1 and FANCA (siFANCA/siNPM1). Cells were transfected for 72 h. Beta-actin (Actβ) served as a loading control. (C) HeLa cells were transfected with siRNA control (siCtrl), siRNA targeting *FANCA* (siFANCA) or siRNA targeting *NPM1* and *FANCA* (siFANCA/siNPM1). Tubulin served as a loading control. (D) Histograms showing normalized mRNA levels for p21 measured by qPCR in HeLa cells transfected with siRNA control (siCtrl) or siRNA targeting *FANCA* (siFANCA) or *FANCA* and *NPM1* (siFANCA/siNPM1) for 72 h. Each point represents an individual experiment. *p<0.05. n = 3. (E) Immunofluorescence of HeLa cells showing the localization of NPM1. Cells were transfected with either siRNA control (siCtrl) or siRNA targeting *FANCA* (siFANCA) for 72 h. (F) Histogram showing the mean intensity of NPM1 in the cytosol in at least 100 cells in 3 independent experiments. Statistical differences were assessed by Student’s t test (*p<0.05). The scale bar is 20 μm. (G) PLA analysis between NPM1 and p21 in HeLa cells transfected with siRNA control (siCtrl) or siRNA targeting FANCA (siFANCA) and treated with or without actinomycin D (ACTD) overnight. Representative images of the different conditions in HCT116 WT cells are shown (left), and quantification in HCT116 WT and in HCT116 *TP53*^*-/-*^ cells are shown (right). Reactions with single primary antibodies were used as negative controls. (H) Western blot showing the levels of p21 and NPM1 in cells transfected with siRNA control (siCtrl), siRNA targeting *FANCA* (siFANCA) or *FANCA* and *NPM1* (siFANCA/siNPM1) for 72 h and then treated with cycloheximide (CHX) for the indicated times. Tubulin served as a loading control. The table shows the percentage of p21 protein remaining after 2 h compared to that at time 0.

We recently demonstrated that FANCA-depleted cells exhibit nucleolar stress, altered ribosome biogenesis and a reduced translational rate [19], and similar findings were reported after FANCI depletion [20]. These abnormalities can lead to increased p21 expression through ATM-independent, p53-dependent and p53-independent mechanisms [34]. Consistently, translation inhibition by puromycin exposure **(Fig 4A)** or NPM1 downregulation **(Fig 4B and 4C)**, known causes of nucleolar and/or ribosomal stress [35,36], also increased p21 expression in the absence of an active p53 protein.

In p53-proficient cells, codepletion of NPM1 and FANCA did not exacerbate the overexpression of p21 induced by the downregulation of each protein **(Fig 4B, left panel)**. In contrast, NPM1 depletion strongly reduced the level of p21 associated with FANCA depletion in a p53-deficient background **(Fig 4B, right panel, and 4C)** without affecting the expression of its encoding RNA **(Fig 4D)**. Nucleolar stress in FA cells was associated with NPM1 displacement in the nucleoplasm ([19], **Fig 4E and 4F**), where it associated with p21, similar to what was observed in cells treated with the nucleolar stressor actinomycin-D ([34], **Fig 4G)**. Finally, using cycloheximide (CHX), a widely recognized assay used to observe intracellular protein degradation and determine the half-life of a given protein in eukaryotes, we demonstrated that the half-life of p21 was significantly extended by NPM1 in FANCA-deficient cells **(Fig 4H)**. We then tested whether codepletion of both MITF and NPM1 would fully restore p21 in FANCA-depleted cells. Surprisingly, although the p21 level was reduced, it remained at a greater level than that in control cells **(S2A Fig)**. This can be due to the lack of complete depletion of FANCA, NPM1 and/or MITF or to the existence of additional mechanisms contributing to p21 overexpression.

This was also observed in an isogenic model of RPE1 cells in which we depleted either NPM1 or MITF in presence or not of p53. Indeed, we showed that depletion of NPM1 was sufficient to reduce the level of p21 independently of p53 status of the cell while MITF was only marginally reducing p21 expression **(S2B Fig)**. Altogether, our observations highlight that p53 and NPM1 cooperate to maintain p21 overexpression. In some situations that could be cell types specific, others transcription factors may sustain an overexpression of p21.

### p21 overexpression in FA cells contributes to S-phase lengthening

**Fig 5:**
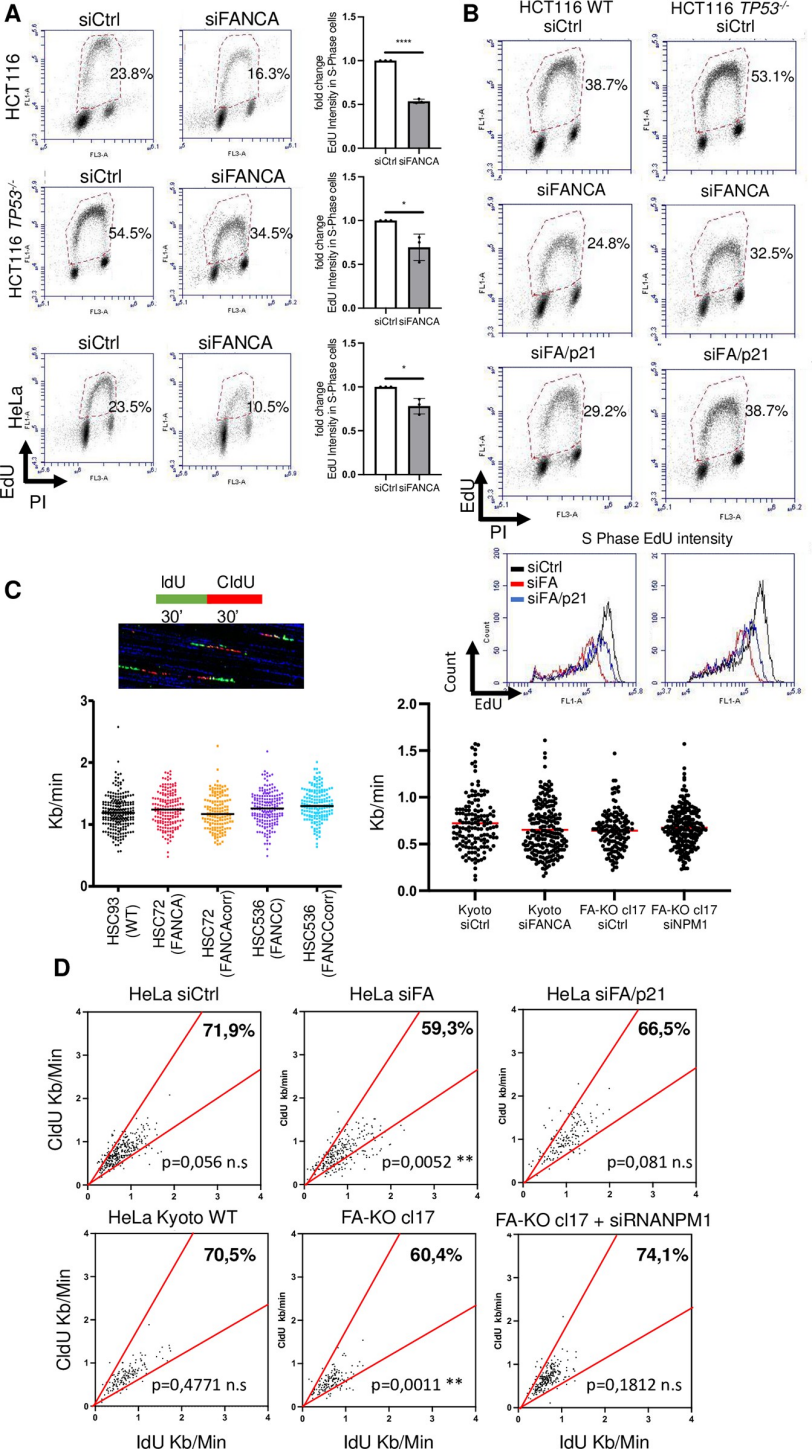
Role of p21 in cell cycle regulation in FANCA-deficient cells. (A and B) Cell cycle analysis of HCT116, HCT116 *TP53*^-/-^ and HeLa cells transfected with siRNA control (siCtrl), siRNA targeting FANCA (siFANCA) or siRNA targeting *FANCA* and *CDKN1A* (siFA/p21). The cell cycle distribution was revealed by PI/EdU costaining and flow cytometry analysis 72 h after transfection. Quantification of the EdU intensity is indicated in the charts in the right panel (n = 3) or lower panel (B). Each point represents an individual experiment. * p<0.05; ****p<0.0001. (C) Analysis of replication fork speed by DNA combing. From left to right, lymphoblastoid cells (N>149); HeLa Kyoto cells, WT or FA-KO clone 17 transfected with siRNA control (siCtrl) or siRNA targeting *FANCA* (siFANCA) or *NPM1* (siNPM1) (N>160). The graph represents one of two experiments showing comparable results. (D) Upper row: Analysis of replication fork asymmetry in HeLa cells transfected with siRNA control (siCtrl), targeting *FANCA* (siFA), or both *CDKN1A* and *FANCA* (siFA/p21) indicated by the IdU/CldU ratio. The percentage of correlated forks is indicated between the red lines that delineate 33% deviation from the expected value of 1. The statistics indicate the difference from the theoretical median value of 1 if the forks move at a constant rate (Mann–Whitney). Lower row: Analysis of replication fork asymmetry in HeLa Kyoto parental cells and in the FANCA knockout clones (clone 17) transfected with siRNA targeting *NPM1* (siNPM1) indicated by the IdU/CldU ratio. The percentage of correlated forks is indicated. The statistics indicate the difference from the theoretical median value of 1 if the forks move at a constant rate (Mann–Whitney).

We previously observed that FANCA-deficient cells exhibited p21-dependent chromatin accumulation of RPA **(Fig 2F)**, suggesting that p21 overexpression participates in the induction of replication stress downstream of FANCA depletion. This prompted us to compare the cell cycle profile of FANCA-proficient cells to that of their FANCA-deficient counterparts. By flow cytometry analysis of EdU/PI double-stained cells, we observed that FANCA depletion was associated with a significant reduction in the fraction of S-phase cells able to incorporate EdU at steady state **(Figs 5A, 5B and S3A Fig)**. Additionally, single-cell analysis revealed a marked decrease in EdU incorporation in the absence of FANCA **(Figs 5A, 5B, S3A and S3B)**. Specifically, the height of the arch formed by the EdU-positive cells was notably lower in the absence of FANCA, indicating a lower level of EdU incorporation (Y-axis) into cells with similar DNA content (i.e., at the same position on the X-axis). This indicate that replication is slowdown in FANCA cells, because of either a reduced replication fork speed or a reduced number of activated origins. p21 downregulation in FANCA-deficient cells did not significantly increase the fraction of the cells in S phase **(Figs 5B and S3A Fig)** but largely rescued EdU incorporation at the single-cell level **(Figs 5B and S3A Fig)**. Our observations suggest that p21 expression in FA cells is not significantly involved in delaying G1/S progression but rather indicates it plays a role in reducing EdU incorporation and therefore to lengthen the S-phase in FANCA-deficient cells.

Furthermore, we observed the restoration of EdU incorporation in FANCA-depleted HeLa cells at the single-cell level following MITF and/or NPM1 depletion, further supporting the involvement of these two proteins in p21 overexpression and the associated abnormalities **(S4 Fig)**.

Previous studies [37] have suggested that excess p21 could influence S-phase length, interfering with the activity of its interactor PCNA [38]. Consistently, in FANCA-depleted cells, p21 coimmunoprecipitated with PCNA **(S5A Fig)** and was associated with chromatin **(S5B Fig)**. In agreement with seminal publications on primary cells from FA patients [39,40], we demonstrated that FANCA depletion leads to an extended S phase. While the S phase is completed in approximately ten hours in WT cells, more than 12 hours are required in the absence of FANCA **(S5C and S5D Fig)**. Reduced EdU incorporation at the single-cell level in cells with the same DNA content and an extended S-phase length could be due to a reduced speed of the replication forks or to alterations in the progressive firing that staggers the progression of replication (replication timing). Therefore, we performed a DNA fiber analysis **(Fig 5C)** to characterize DNA replication at the single-molecule level in patient-derived *FANCA*^-/-^ or *FANCC*^-/-^ lymphoblasts and in FANCA-depleted or KO HeLa cells **(Figs 5C and S5E)**. Regardless of the cell type and p53 background, DNA combing analysis revealed no difference in replication fork speed between FANC pathway-proficient and FANC pathway-deficient cells. Moreover, depletion of NPM1, which strongly reduces p21 expression **(Fig 4C)**, did not affect the speed of the replication fork in FANCA-deficient cells **(Fig 5C)**. Therefore, FANCA depletion and the associated p21 overexpression do not affect replication fork speed.

Previous work suggested that FANCM, the upstream FANCA regulator [41] and ATR partner [42,43], opposes the rate of DNA chain elongation (hence, its loss results in an increased fork speed), ensuring constant progression of the replication forks over time and limiting the accumulation of single-stranded DNA (the RPA substrate) [44]. FANCA deficiency did not affect fork speed **(Fig 5C)** but it did increase RPA accumulation in chromatin **(Fig 2F)** (hence increasing the number of single-stranded DNA regions). This finding prompted us to ask whether, in the absence of FANCA, the fork moves irregularly, as reported in FANCM-depleted cells [44]. Thus, we reconsidered our analysis of DNA fibers stained with IdU and CldU. While the average length of each (IdU + CldU) trait (used to calculate replication fork speed) was similar in FANCA-competent and -deficient cells **(Fig 5C)**, the tract length of each analogue within of the same replication fork resulted more often nonhomogeneous in the absence of FANCA, revealing increased asymmetry in FANCA-deficient cells **(Fig 5D).** Importantly, p21 or NPM1 depletion normalized fork symmetry **(Fig 5D)**.

Thus, it appears that p21 overexpression in FA cells influences the steady progression of a replication fork. Given that in FA cells S-phase progression is delayed (Fig 5A) despite normal replication fork speed **(Fig 5C)**, we investigated whether loss of FANCA function contributes to alterations in the regulation of CDKs activities and CDT1 expression/chromatin accumulation, key mediators of the timely regulated progression of the replication timing program. Notably, in FANCA-deficient cells and in a p21-dependent manner, CDK activities appeared reduced while CDT1 expression is upregulated **(S5B and S5F Fig)**.

The previous observations support the possibility that the progressive firing of common clusters of replication origins that marks the progression of the S-phase could be impaired in FA cells as consequence of p21-mediated CDKs activities downregulation. Increased asymmetry indicates that overexpression of p21 impacts the steady progression of a replication fork, either per se or because its increased association with PCNA amplifies the consequences of other nuisances affecting replisome advancement. However, regardless of the cause of the observed asymmetry, the reduced level of EdU incorporation in a single cell we observed by flow cytometry analysis in FA-deficient cells cannot be due to a change in the speed of the replication fork speed, as assessed by DNA combing. Thus, we speculate that reduced EdU incorporation and extended S-phase in FA cells are possible consequences of a delayed progression of the replication-timing program, known to be dependent on timely regulation of CDK activity and CDT1 expression/chromatin accumulation. Accordingly, in FANCA-deficient cells and in a p21-dependent manner, CDK activity is downregulated whereas CDT1 expression is upregulated **(S5B and S5F Fig)**.

### Increased intracellular levels of p21 and reactive oxygen species jointly contribute to replication stress and genetic instability

**Fig 6:**
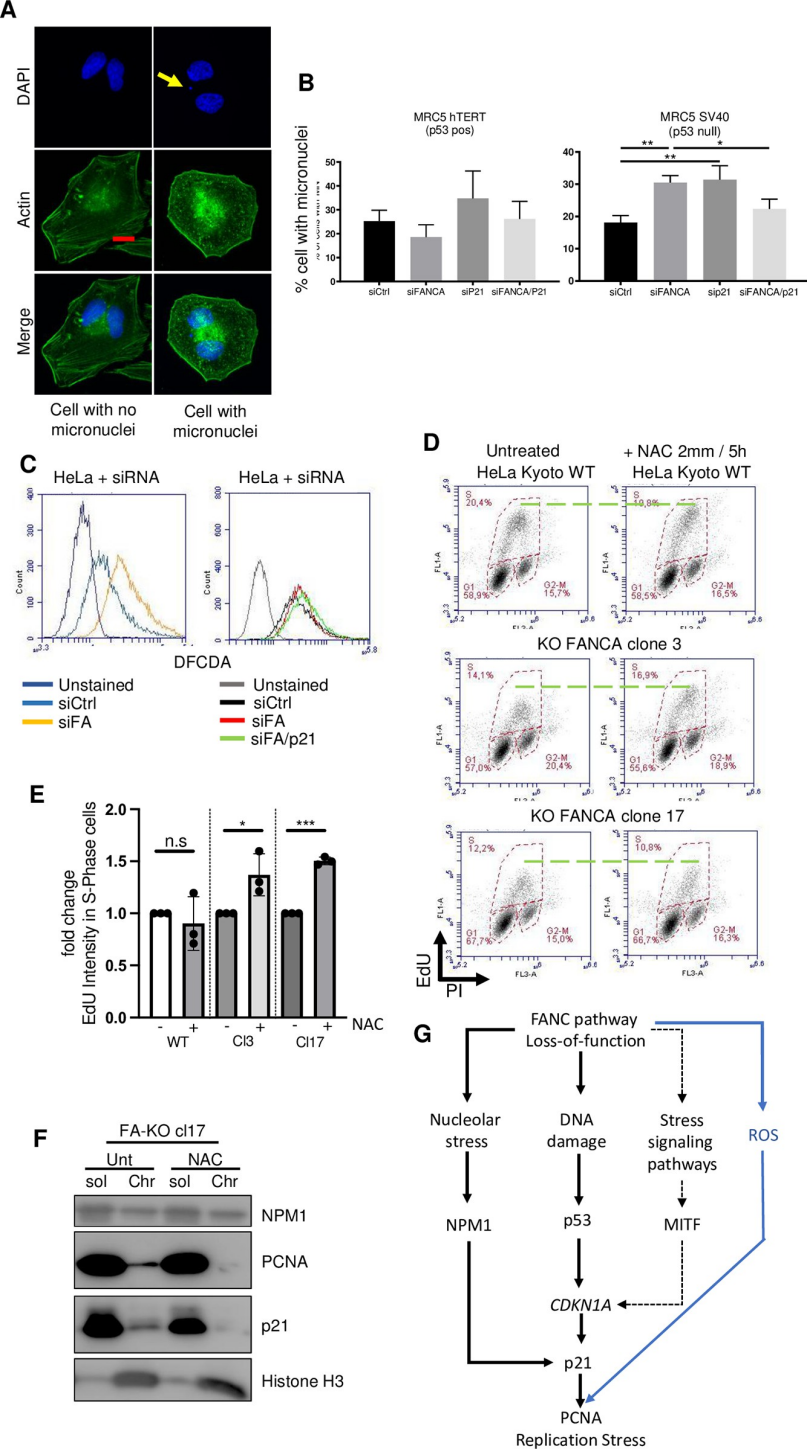
ROS participate in p21-induced replication stress. (A) Immunofluorescence showing typical cells analyzed for micronuclei. Cells were transfected with siRNA for 48 h and then incubated for 18 h with cytochalasin B (2 μg/ml) to prevent the formation of the actin filament ring. Only binucleated cells were counted. (B) Histograms showing the number of micronuclei in cells transfected with the indicated siRNA. At least 50 cells were counted. Statistical differences are indicated (n = 3; * p<0.05; **p<0.01). (C) ROS levels in cells transfected with siCtrl or siRNA targeting *FANCA* or both *FANCA* and *CDKN1A* (siFA/p21). Intracellular ROS were measured by flow cytometry after incubation with 5 μM CM-H2DCFDA for 15 min. (D) Cell cycle analysis of HeLa Kyoto parental cells and FANCA knockout clones 3 and 17 after treatment with 2 mM NAC for 5 h. Cell cycle distribution was revealed by PI/EdU costaining and flow cytometry analysis. Dashed lines indicate the highest level of EdU staining. The percentage of cells in the different cell cycle phases is indicated. (E) Histograms showing the level of EdU intensity in the cell lines analyzed in F. Each point represents an individual experiment. * p<0.05; *** p<0.001. (F) Western blot showing the levels of soluble (sol) and chromatin-bound (Chr) proteins (NPM1, PCNA, p21) in HeLa Kyoto FANCA-KO clone 17 cells exposed or not to N-acetyl cystein (NAC) (2 mM). Histone H3 served as a loading control. (G) Model summarizing the findings presented in this manuscript.

Maintenance and control of the physiological level of p21 are crucial for cellular health, but this process depends on the cellular and genetic background [45]. Uncontrolled expression of p21, either inappropriately downregulated or upregulated, can affect genetic stability. Because loss of genetic stability is a hallmark of FA cells and a diagnostic criterion for FA, we questioned the consequences of p21 overexpression in FANCA-depleted cells in terms of genomic stability. Transient siRNA-mediated depletion of FANCA led to a significant increase in the frequency of cells harboring postmitotic micronuclei, a widely accepted indicator of chromosomal abnormalities, only in the absence of functional p53 **(Fig 6A and 6B)**. Downregulation of p21 in these cells rescued their genetic instability caused by FANCA depletion. Thus, in p53- and FANCA-deficient cells, p21 overexpression contributes to genetic instability. In contrast, when p53 is intact, cell death or senescence pathways may serve as a protective mechanism that suppresses this p21-dependent effect [46,47].

Beyond endogenous nuclear stress, FA cells experience chronic oxidative stress, which amplifies their genetic instability along with other phenotypic traits. Notably, replication stress can also be generated by oxidative stress. Accordingly, FANCA depletion/knockout was associated with increased levels of intracellular ROS, as determined by flow cytometry analysis of CM-H2DCFDA-stained cells **(Figs 6C and S6A)**. Depletion of p21 in FANCA-deficient cells did not significantly alter their intracellular ROS levels **(Figs 6C and S6B)**. Therefore, the overexpression of p21 and its associated consequences on replicative stress are not responsible for the intracellular ROS accumulation observed in FANC-deficient cell lines. However, reducing the intracellular level of ROS rescued the EdU incorporation level in FANCA-deficient cells **(Figs 6D, 6E, and S6C)**. Unexpectedly, the intracellular pro-oxidant status of FANCA-deficient cells indeed seems to be responsible for the high chromatin accumulation of PCNA and p21 **(Fig 6F)**.

Thus, our observations highlight the joint contribution of p21 overexpression and ROS overproduction to induce the replicative stress and the genetic instability that characterize FA cells **(Fig 6G)**.

## Discussion

This study revealed a cooperative network that connects two seemingly unrelated pathways downstream of FANC pathway dysfunction. The p21 upregulation and increased intracellular ROS levels appear to be jointly required in FA cells to disrupt EdU incorporation during unperturbed DNA synthesis without altering replication fork speed, resulting in S-phase lengthening and genetic instability **(Fig 6G)**. Remarkably, p21 overexpression in FANC pathway-deficient cells does not influence intracellular ROS level, which, in turn, does not appear to contribute significantly to p21 overexpression. p21 overexpression. However, ROS may mediate the association of p21 with PCNA/chromatin, possibly through the induction of oxidative lesions on DNA and/or chromatin. This association could be necessary for the inhibitory effect of p21 on timely S-phase progression, possibly through the modulation of CDK activity. Downregulation of p21 in FANCA-deficient cells is sufficient to rescue their spontaneous chromosomal fragility and hematopoietic stem cell clonality [22], similar to the effects observed after normalization of intracellular ROS levels in FA cells. Thus, increased ROS and p21 overexpression are intrinsically linked and jointly responsible for altered S-phase/replication stress and genomic instability, which are hallmarks of Fanconi anemia cells **(Fig 6G)**.

Encoded by the CDKN1A gene, p21 is a master regulator of cellular homeostasis, influencing essential physiological processes such as the G1 and G2 checkpoints, S-phase progression, DNA replication and repair, transcription, differentiation, senescence, and apoptosis, all of which are altered in FA cells. Therefore, tight regulation of p21 expression in terms of quantity, timing, and intracellular localization is crucial to avoid pathological consequences for the cell and the organism [45]. However, (over)expression of p21 can result in paradoxical outcomes, including mediating senescence evasion and increasing cell proliferation [45,48]. Indeed, whereas p21 appears to be necessary for the maintenance of the self-renewal capability of leukemia stem cells [49], its upregulation appears to be involved in the hematopoietic stem cell attrition that characterizes several BMF syndromes [5], as reported in FA [22]. Inhibition of p21 or p53 may lead to an increase in genomic instability, even though in vitro data suggest that it may be sufficient to restore a pool of HSCs.

p21 expression and activity are dependent on several transcription factors and on its interactions with various proteins [48,50–53], some of which are deregulated in FA, including p53 [21–23], MITF [18,31], c-Myc [10], NF-kB [54,55] and NPM1 [34]. Consistently, we demonstrated a role for p53, MITF and NPM1.

Moreover, we recently reported that FA cells exhibit nucleolar stress similar to what has been described in other ribosomopathies, including nucleolus deformation and defects in translation during ribosome biogenesis. A direct consequence of this nucleolar stress is the increased abundance of NPM1 in the nucleoplasm, which is usually contained in the nucleolus and therefore bound to p21. The origin of the nucleolar stress remains under investigation since a direct effect of DNA damage has been ruled out [19].

Whether p21 overexpression is key in shifting the normal homeostasis of the hematopoietic system to the BMF and, later, to the expansion of preleukemic clones **(S6D Fig)** remains to be elucidated. Indeed, leukemic progression in FA cells is associated with two main events: genetic inactivation (translocations, deletions, or mutations) of the RUNX1/AML1 locus [56], a master regulator of the differentiation program of the cells, and amplification of the 1q/MDM4 locus, which leads to overexpression of the p53 suppressor MDM4, allowing downregulation of basal p53 activation without the need for inactivating mutations in the tumor suppressor [57].

Additionally, our observations potentially offer new avenues to “normalize” the level of p21, acting on MITF or NPM1 expression rather than inhibiting it. The present study has potential implications not only for FA patients but also for patients suffering from cancers with p53 mutations and increased p21 levels.

## Materials and methods

### Cell lines, culture conditions and treatments

HeLa, HeLa Kyoto (parental and FANCA-KO), HCT116 (WT and p53-KO), and MRC5 (SV40 and hTERT) cells were grown in DMEM supplemented with 10% fetal bovine serum, 1 mM sodium pyruvate, 100 U/ml penicillin and 100 μg/ml streptomycin (all from Invitrogen). RPE1 cells were grown in F12 medium with 10% fetal bovine serum, 1 mM sodium pyruvate, 100 U/ml penicillin and 100 μg/ml streptomycin.

K562 (parental and FANCA-KO, a generous gift from Prof J Corn, ETH Zürichand) and the EBV-immortalized lymphoblastoid cell lines HSC72 (FANCA deficient) and HSC536 (FANCC deficient) and their respective corrected counterparts HSC72corr and HSC536corr, along with HSC93 (wild type), HSC99 (FANCA deficient), EGF004 (FANCG), EGF070 (FANCG), and GM16756 (FANCD2) were grown in RPMI 1640 supplemented with 13% fetal bovine serum, 100 U/ml penicillin and 100 μg/ml streptomycin (all from Invitrogen).

All cells were cultivated at 37°C under a 5% CO_2_ atmosphere. For some experiments, the level of O_2_ was controlled at 5%.

Treatments with cycloheximide (CHX), which was prepared as a 1000X stock solution in DMSO, were performed at the indicated doses and times. Mitomycin C (200 ng/mL), hydroxyurea (1 mM) and aphidicolin (0.2 μM) were prepared in H_2_O and added for 18 hours. NAC, prepared at a 2 M stock concentration, was added directly to the cell media at a 2 mM concentration for 5 h. ATM and/or the ATR inhibitors KU-55933 (Abcam) (10 μM) and VE-822 (Sellchem) (100 nM) were added to the culture media for 18 hours after transfection. PFTα and puromycin were purchased from Sigma.

### Generation of FANCA knockout cells

HeLa FANCA-KO cells were generated from HeLa Kyoto cells. Cells were plated in a 12-well plate and cotransfected with the FANCA targeting guide RNA (5’-CACCTAAAGCTTCTTAAGATATC-3’) clone in the PX458 vector using Lipofectamine 2000 reagent. Two days after transfection, the cells were replated in a 14 cm dish and selected with puromycin (1 μg/ml). Selected clones were expanded and screened by Western blotting.

### Transfection (siRNA and plasmids)

The control siRNA (5′-CGUCGACGGAAUACUUCGA-3′) was purchased from Eurogentec. siRNAs against MITF, p21 and FANCA were purchased from Dharmacon. All siRNA transfections were performed using the calcium chloride protocol. Briefly, 30 min prior to transfection, the cell culture medium was replaced with medium without antibiotics. siRNA (20 nM) was diluted in CaCl_2_ (125 mM) and H_2_O and mixed with an equal volume of HBSP buffer (50 mM HEPES, 280 mM NaCl, 15 mM Na_2_HPO_4_, 10 mM KCl, pH 7.04), and the experiments were conducted 72 h later.

### Antibodies

We used the following antibodies: mouse anti-FANCD2 (Santa Cruz; sc-20022), rabbit anti-p21 (Cell Signaling Technology; CST2947), FITC-conjugated anti-p21 (Cell Signaling Technology; CST5487), rabbit anti-phospho-CDK substrate (Cell Signaling Technology; CST9477), mouse anti-γH2AX (Millipore; #05–638), rabbit anti-histone H3 (Abcam; ab1791), mouse anti-tubulin (Sigma, TS168), mouse anti-p53 (Millipore; OP43T), goat anti-actinB (Abcam; ab8229), mouse anti-PCNA (Santa Cruz; sc-56), mouse anti-R-loop (Sigma; MABE1095), rabbit anti-FANCA (Bethyl; A301-980), mouse anti-NPM1 (Thermo Fisher; #32–5200), and secondary HRP-conjugated goat anti-mouse (Tebu), donkey anti-rabbit (Tebu) and donkey anti-goat (Tebu) antibodies. The isotype antibodies mouse IgG1 and IgG2a,b and rabbit IgG were obtained from Dako.

### qPCR analysis

Total RNA was isolated using the Maxwell RSC simplyRNA Cell Kit (Promega, AS1390) and reverse-transcribed using the RevertAid First Strand cDNA Synthesis Kit (Thermo Scientific, K1622). qRT–PCR was performed using Maxima SYBR Green/ROX qRT–PCR Master Mix (Thermo Scientific, K0222) and a CFX96 Touch Real-Time PCR Detection System (Bio-Rad). PCR was performed on cDNA using DreamTaq Green DNA Polymerase (Thermo Scientific, EPO713). The following primers (Eurogentec) were used for qRT–PCR: Pan-*MITF* (forward, 5′-CGAGCTCATGGACTTTCCCTTA-3′ and reverse, 5′-CTTGATGATCCGATTCACCAAA-3′), *actin* (forward, 5′-GACGGCCAGGTCATCACTATTG-3′ and reverse, 5′-AGGAAGGCTGGAAAAGAGCC-3′), and *CDKN1A* (forward, 5′-AGGTGGACCTGGAGACTCTCAG-3′ and reverse, 5′-TCCTGGAGAAGATCAGCCG-3′).

### Western blot analysis

The collected cells were disrupted in lysis buffer [50 mM Tris-HCl pH 7.9, 40 mM NaCl, 1 mM MgCl_2_, 0.1% SDS and 1% benzonase (Novus)] supplemented with protease and phosphatase inhibitors (Roche). After 20 min of incubation at room temperature, the protein concentration was determined using a BCA assay (Thermo Fisher Scientific), and the samples were combined with 4X Laemmli buffer containing β-mercaptoethanol and denatured by boiling. The proteins (20–30 μg) were separated by SDS–PAGE. All Western blot quantifications were performed using densitometry measures and ImageJ software.

### Protein immunoprecipitation

The proteins were extracted using NETN buffer (150 mM NaCl, 50 mM Tris-HCl (pH 8.0), 0.5–1% NP-40 and 1 mM EDTA) and sonicated to fragment the DNA. The extracts were precleared using a nonspecific antibody for 1 h at 4°C. The cleared extracts were added to magnetic Protein G beads (Millipore) that were coated with a specific antibody, and the extracts and beads were incubated overnight at 4°C. Control immunoprecipitations were conducted using a nonspecific isotype control antibody under the same conditions. After four washes in NETN buffer, the immune complexes were eluted by boiling for 5 min in 35 μl of 2× Laemmli buffer and were denatured by boiling for another 5 min in the presence of 1 μl of 1 M β-mercaptoethanol.

### Cell fractionation

The cell pellets were resuspended in solution A (HEPES pH 7.9 10 mM, MgCl_2_ 1.5 mM, sucrose 12%, glycerol 10%, DTT 1 mM, protease and phosphatase inhibitors) supplemented with 0.1% Triton X-100. The samples were incubated for 5 min on ice. The samples were centrifuged for 4 min at 1300 × g, after which the soluble proteins (S1) were collected and stored. The nuclei were resuspended and incubated in solution B (3 mM EDTA, 0.2 mM EGTA, 1 mM DTT, and protease and phosphatase inhibitors) for 10 min and then centrifuged for 4 min at 1700 g, after which the soluble nuclear proteins (S2) were collected. After a wash in solution B, the chromatin (P2) was pulled down by centrifugation at 16 000 × g, resuspended in lysis buffer supplemented with 2% benzonase and then incubated at RT for 15 min. The S1 and S2 fractions were mixed with Laemmli 4× buffer. All of the fractions were denatured by boiling and analyzed by Western blotting. Whole-cell extracts (WCEs) were obtained with the standard protocol described above from a small portion of the cells that were used for fractionation.

### Flow cytometry analysis

To determine p21 levels, cells were harvested and washed once in cold PBS before centrifugation at 150 g for 5 min. Cells were fixed in 2% PFA for 10 min and then permeabilized with PBS containing 0.2% Triton X-100. After two washes, the cell pellets were resuspended in PBS containing 3% bovine serum albumin (BSA) with an Alexa Fluor 488-conjugated anti-p21 antibody diluted 1:100 and incubated for 1 h at room temperature in the dark. After two washes with cold PBS, the cells were incubated in propidium iodide (PI, 3 μg/mL) for 15 min and then washed with PBS containing 3% BSA. After two washes, the cells were resuspended in PBS before analysis.

For cell cycle analysis, 10 μM EdU was incubated with the cells for 30 min. The cells were then collected and fixed with 70% ice-cold ethanol. The cells were then rehydrated, washed with cold PBS and centrifuged at 500 × g for 5 min. Detection was performed via a click-IT reaction according to the manufacturer’s protocol (Invitrogen).

S-phase duration experiments were performed according to [58] with minor modifications. Briefly, the cells were pulsed for 30 min with EdU and then chased with thymidine and nocodazole for the indicated time, followed by a second pulse of BrdU for 30 min. The cells were subsequently fixed with 70% ice-cold ethanol. The cells were then rehydrated, washed with cold PBS and centrifuged at 500 × g for 5 min. EdU was detected as described above, and BrdU was detected as previously described [59].

For ROS measurement, CM-H2DCFDA (Thermo Fisher), dissolved in DMSO, was added to DMEM without phenol red and incubated for 15 minutes at 37°C (at a final concentration of 5 μM). The cells were subsequently rinsed with PBS, trypsinized, collected and rinsed twice with PBS.

The cells were analyzed using a C6+ cytometer with CFlow software (Accuri) with appropriate excitations and bandpass filters.

### Immunofluorescence, proximity ligation assay, and micronuclei measurement

Cells were grown on glass coverslips and fixed in 4% formaldehyde for 10 min at room temperature before permeabilization in 0.5% Triton X-100 for 5 min. After blocking with 3% BSA in PBS containing 0.05% Tween 20, the cells were stained for 1 h in blocking solution with the indicated antibodies. Primary antibody detection was achieved by incubation with anti-rabbit or anti-mouse Alexa Fluor 315-, 488- or 594-conjugated secondary antibodies (Invitrogen; 1/1000) for 1 h at room temperature. Slides were mounted in DAKO mounting medium supplemented with DAPI (Sigma) and examined at a magnification of 63× by fluorescence microscopy (Zeiss Axio Observer Z1). Immunofluorescence images were quantified by densitometry analysis using ImageJ software. For each nucleus, two ROIs outside the low-intensity DAPI region were defined as the nucleoli.

For the proximity ligation assay, cells grown on glass coverslips were fixed with 4% paraformaldehyde (Sigma) for 10 min at room temperature and then permeabilized in PBS containing 0.05% Tween 20 and 0.5% Triton X-100 (both from Sigma) for 5 min. After being blocked in PBS containing 2% BSA, the cells were incubated in a solution of primary antibody diluted in PBS with 2% BSA for 2 h and then incubated with a PLA probe for 30 min at 37°C. Next, ligation and amplification were performed following the manufacturer’s protocol.

For micronuclei detection, after 48 h or siRNA transfection, the cells were incubated in cytochalasin B (2 μg/ml; Sigma) for an additional 24 h before fixation to score the micronuclei in the binucleated cells.

Images were captured with an ORCA-ER camera (Hamamatsu). The microscope and camera parameters were adjusted for each series of experiments to avoid signal saturation. Image processing and analysis were performed using FiJi software (https://fiji.sc/).

### DNA combing

DNA combing was performed as previously described [60]. Briefly, cells were pulse-labeled for 30 min with 20 μM final concentration of IdU followed by 30 min of CldU (100 μM final concentration). The cells were collected and embedded in 2% low melting agarose plugs and subsequently incubated for 48 hr at 42°C in PK buffer (1% SDS, 0.25 EDTA and 1 mg/ml proteinase K). Agarose plugs were heated at 68°C for 15 min in 1X TE containing β-agarase I buffer (NEB, M0392) and then at 42°C for 48 hr in the presence of β-agarase I (NEB, M0392) and for 30 min in 0.25 M MES, pH 5.5. DNA present in the melted β-agarase-digested agarose was stretched onto in-house silanized coverslips at a constant speed (300 μm/sec) with an automated combing device (FiberComb, Genomic Vision). Slides were denatured in 1 N NaOH for 5 min at RT, rinsed in 1X PBS at 4°C for 5 min and dehydrated in increasing concentrations of ethanol (70%-85% and 100%) for 5 min each at RT. The slides were then incubated with BlockAid blocking solution (Thermo Fisher B10710) for 1 h at 37°C, followed by incubation with mouse anti-BrdU-FITC (347580, BD Biosciences) and rat anti-BrdU (Abcam, ab6326) antibodies. Next, the samples were washed in 0.5 M NaCl, 20 mM Tris (pH 7.8), and 0.5% Tween 20 for 6 min at RT. The samples were then incubated for 30 min at 37°C with goat anti-mouse Alexa Fluor Cy55 secondary antibodies (Abcam, ab6947) together with goat anti-rat Alexa Fluor 555 secondary antibodies (Invitrogen, A21434). The slides were washed 3 times for 5 min each in 1X PBS and incubated for 30 min at 37°C with mouse anti-ssDNA (Millipore, MAB3034). After washing in 1X PBS, the slides were incubated for 30 min at 37°C with a goat anti-mouse AF488 (Invitrogen A-11029) antibody, washed, and mounted with VectaShield (Vector Laboratories, H-1000).

Pictures were taken with an Axio Imager. A Z2 (Zeiss) was equipped with a 63X objective lens (PL APO, NA 1.4 Oil DIC M27) connected to a charge-coupled device camera (Cool-SNAP HQ2; Roper Scientific), and MetaMorph software (Roper Scientific) was used for image acquisition.

To calculate the replication speed, only IdU tracks flanked by CldU tracks present in intact fibers, established by DNA counterstaining, were measured. Tracks ending at the same point as the DNA counterstained fiber were omitted. To determine the length in kb/min, the ratio between the lengths of the IdU + CldU tracks divided by the labeling time in minutes was calculated using ImageJ (FIJI) software. Statistical analysis of the median distributions was performed with the nonparametric Mann–Whitney rank sum test using GraphPad Prism 8 software.

### Statistical analysis

Significance was determined by using a two-tailed t test. P values <0.05 were considered significant. All graphs and statistics were generated using GraphPad Prism version 8 or above.

## Supporting information

S1 Fig p21 is increased in FANCA cells independent of p53. (A) Western blot showing the knockout of FANCA in two HeLa Kyoto clones. (B) Western blot showing the level of p21 protein in FANCA-deficient cells (HSC72) and their corrected counterpart (HSC72corr) after treatment with the p53 inhibitor pifhithrin **α** (PFT**α**). The cells were treated with the indicated dose overnight. Red Ponceau staining of the membrane served as a loading control. (C) p21 expression level according to DNA content. The indicated cells were transfected with either a siRNA control (siCtrl) or a siRNA targeting FANCA (siFANCA) for 72 h. The permeabilized cells were incubated (middle and right panels) or not (left panel) with an antibody specific for p21 coupled with FITC and PI before analysis by flow cytometry.
(TIF)

S2 Fig MITF and NPM1 participate in p21 overexpression. (A) Western blot showing the effect of NPM1 and MITF codepletion along with FANCA on p21 levels in HeLa cells. (B) Western blot showing the effect of NPM1 and MITF codepletion along with FANCA on p21 levels in RPE cells having or not TP53.
(TIF)

S3 Fig Cell cycle abnormalities in FANC cells. (A) Cell cycle analysis of HeLa cells transfected with siRNA control (siCtrl), targeting FANCA (siFANCA) or FANCA and p21 (siFA/p21). The cell cycle distribution was revealed by PI/EdU costaining and flow cytometry analysis 72 h after transfection. The quantification of the EdU intensity is presented in the last panel on the right of the row. (B) Cell cycle analysis of WT or FANCA KO Clone 3 and Clone 17 HeLa Kyoto cells. The cell cycle distribution was revealed by PI/EdU costaining and flow cytometry analysis. Quantification of the EdU intensity is indicated in the charts in the right panel (n = 3). Each point represents an individual experiment. * p<0.05.
(TIF)

S4 Fig MITF and NPM1 contribute to cell cycle abnormalities. Cell cycle analysis of HeLa cells transfected with the indicated siRNAs. The cell cycle distribution was revealed by PI/EdU costaining and flow cytometry analysis 72 h after transfection. The quantification of the EdU intensity is presented in the lower panel.
(TIF)

S5 Fig FANC cells have an extended S phase. (A) Coimmunoprecipitation of endogenous PCNA with p21. Immunoblots were performed with antibodies against PCNA or p21. (B) Western blot showing the levels of CDT1 and p21 in the chromatin in cells transfected with the indicated siRNAs. (C) Flow cytometry was used to determine the S-phase duration. Asynchronous cells were pulse-labeled for 30 min with 10 μM EdU, washed, and then pulse-labeled again with 100 μM BrdU for 30 min. Samples received the same two pulses but were separated by a thymidine chase period lasting 2 h, 4 h, 6 h or 9 h. Nocodazole was added to prevent progression to the next cycle. Note that the number of double-positive cells (top right quadrant of the middle panel) decreased over time (between 30 min and 9 h). Linear regression of the fraction of EdU+ BrdU+ cells among EdU+ cells over time was used to determine DNA synthesis time as the time when the regression line crossed the x-axis (bottom panel). (D) Flow cytometry was used to determine the S-phase duration. Asynchronous cells were pulse-labeled with 10 μM EdU and then with 100 μM BrdU for 30 min. Samples received the same two pulses but were separated by a thymidine chase period lasting 2 h, 4 h, 6 h, and 9 h. Nocodazole was added to prevent progression to the next cycle. Note that the number of double-positive cells (top right quadrant of the middle panel) decreased over time (between 30 min and 9 h). (E) Analysis of replication fork speed by DNA combing in HeLa cells transfected with siRNA control (siCtrl; n = 174) or siRNA targeting FANCA (siFANCA; n = 314). The graph represents one of two experiments showing comparable results. (F) Western blot showing the levels of phospho-CDK substrates in HeLa cells transfected with siRNA control (siCtrl), targeting FANCA (siFANCA) or targeting both FANCA and p21 (siFANCA/sip21). Tubulin was used as a loading control.
(TIF)

S6 Fig ROS impact p21 protein levels in FANCA-deficient cells. (A) ROS levels in HeLa Kyoto WT and FANCA-KO cell clones 3 and 17 transfected with the indicated siRNA. (B) ROS levels in HeLa Kyoto FANCA-KO cell clones 3 and 17 transfected with the indicated siRNA. Intracellular ROS were measured after incubation with 5 μM CM-H2DCFDA for 15 min. (C) Cell cycle analysis of HeLa Kyoto parental cells and the FANCA knockout clone (clone 3) after 5 days at either 20% or 5% oxygen, as indicated. The cell cycle distribution was revealed by PI/EdU costaining and flow cytometry analysis. (D) Proposed model for the progression from healthy hematopoiesis to leukemia in FA patients.
(TIF)

S1 Table Numerical values supporting each graph. (XLSX)
