# Supplementary figures and images for: Contribution of p53-dependent and -independent mechanisms to upregulation of p21 in Fanconi anemia
Source: PLoS Genet. 2024 Nov 7;20(11):e1011474. doi: 10.1371/journal.pgen.1011474 (PMC11575784; doi:10.1371/journal.pgen.1011474)

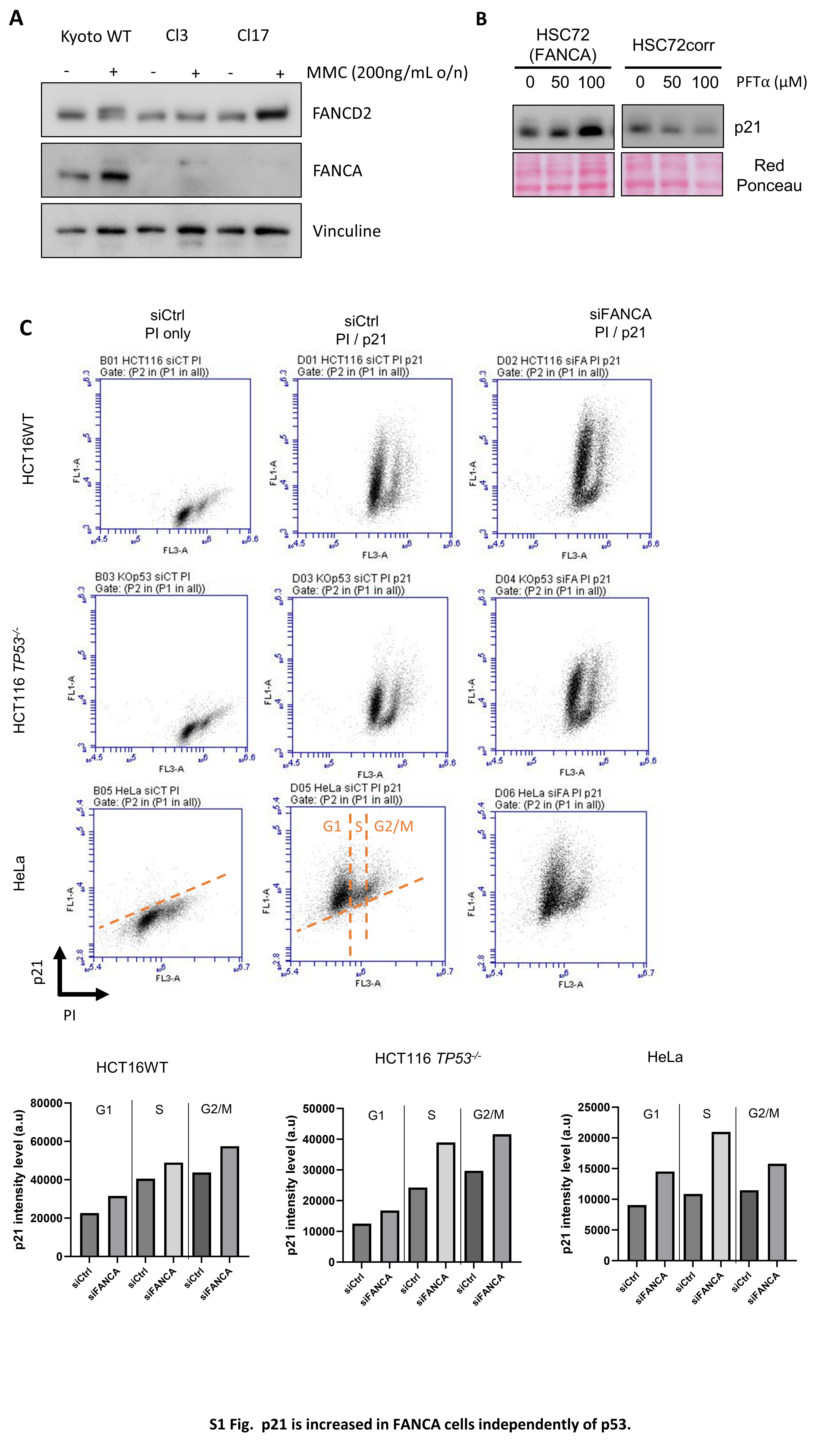

Supplement: S1 Fig — (A) Western blot showing the knockout of FANCA in two HeLa Kyoto clones. (B) Western blot showing the level of p21 protein in FANCA-deficient cells (HSC72) and their corrected counterpart (HSC72corr) after treatment with the p53 inhibitor pifhithrin α (PFTα). The cells were treated with the indicated dose overnight. Red Ponceau staining of the membrane served as a loading control. (C) p21 expression level according to DNA content. The indicated cells were transfected with either a siRNA control (siCtrl) or a siRNA targeting FANCA (siFANCA) for 72 h. The permeabilized cells were incubated (middle and right panels) or not (left panel) with an antibody specific for p21 coupled with FITC and PI before analysis by flow cytometry. (TIF) [file pgen.1011474.s001.TIF]

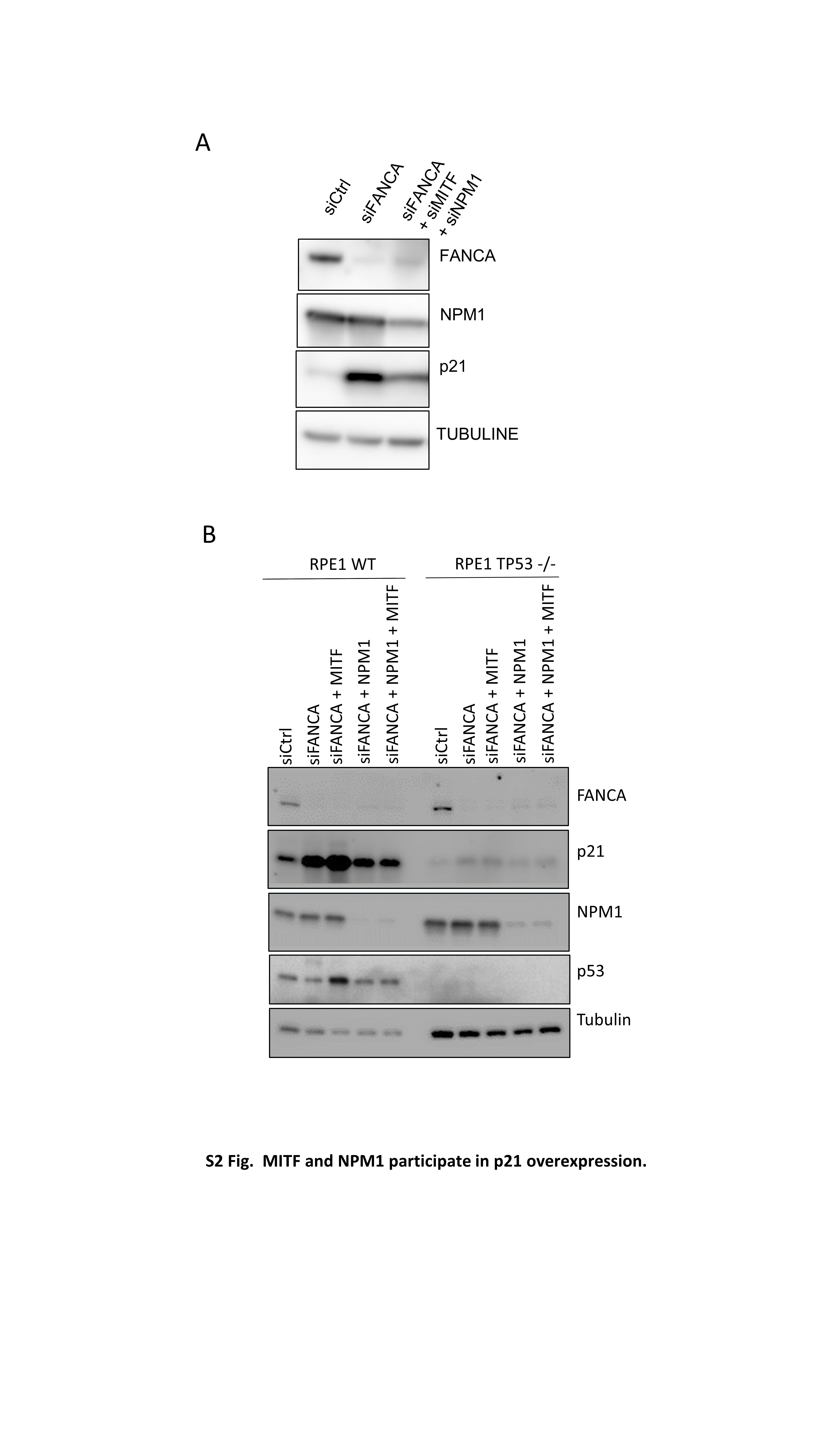

Supplement: S2 Fig — (A) Western blot showing the effect of NPM1 and MITF codepletion along with FANCA on p21 levels in HeLa cells. (B) Western blot showing the effect of NPM1 and MITF codepletion along with FANCA on p21 levels in RPE cells having or not TP53. (TIF) [file pgen.1011474.s002.TIF]

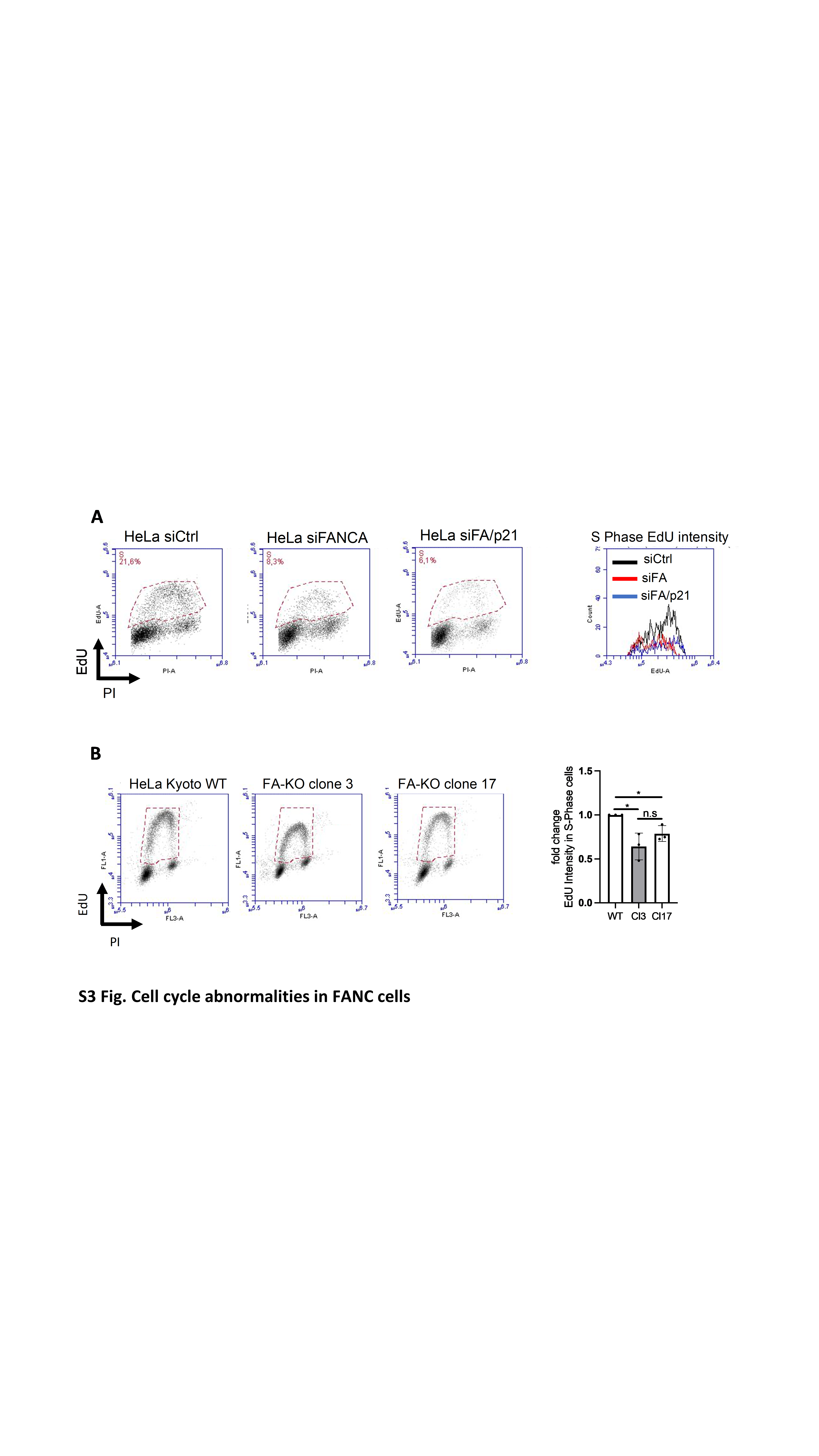

Supplement: S3 Fig — (A) Cell cycle analysis of HeLa cells transfected with siRNA control (siCtrl), targeting FANCA (siFANCA) or FANCA and p21 (siFA/p21). The cell cycle distribution was revealed by PI/EdU costaining and flow cytometry analysis 72 h after transfection. The quantification of the EdU intensity is presented in the last panel on the right of the row. (B) Cell cycle analysis of WT or FANCA KO Clone 3 and Clone 17 HeLa Kyoto cells. The cell cycle distribution was revealed by PI/EdU costaining and flow cytometry analysis. Quantification of the EdU intensity is indicated in the charts in the right panel (n = 3). Each point represents an individual experiment. * p<0.05. (TIF) [file pgen.1011474.s003.TIF]

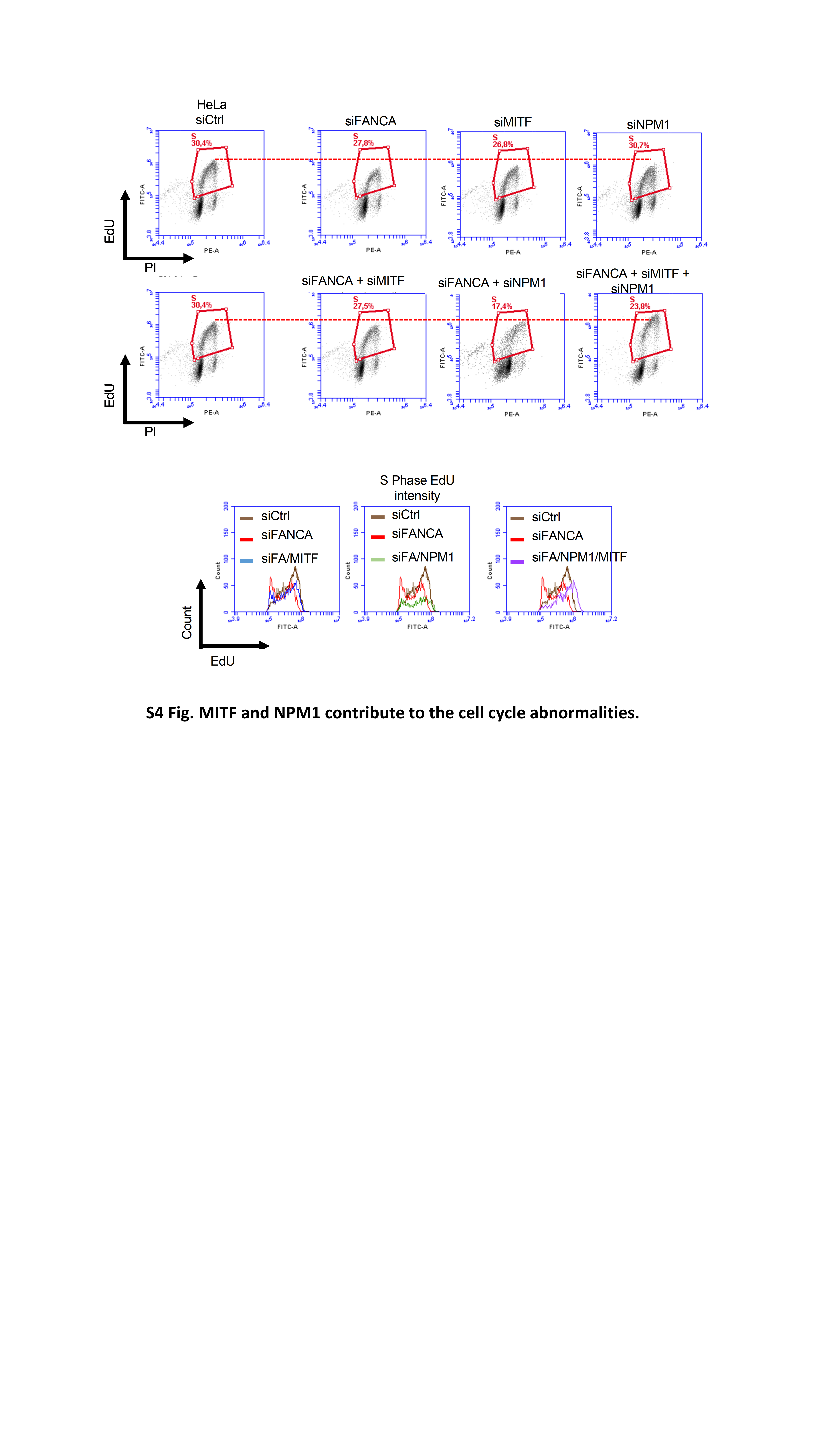

Supplement: S4 Fig — Cell cycle analysis of HeLa cells transfected with the indicated siRNAs. The cell cycle distribution was revealed by PI/EdU costaining and flow cytometry analysis 72 h after transfection. The quantification of the EdU intensity is presented in the lower panel. (TIF) [file pgen.1011474.s004.TIF]

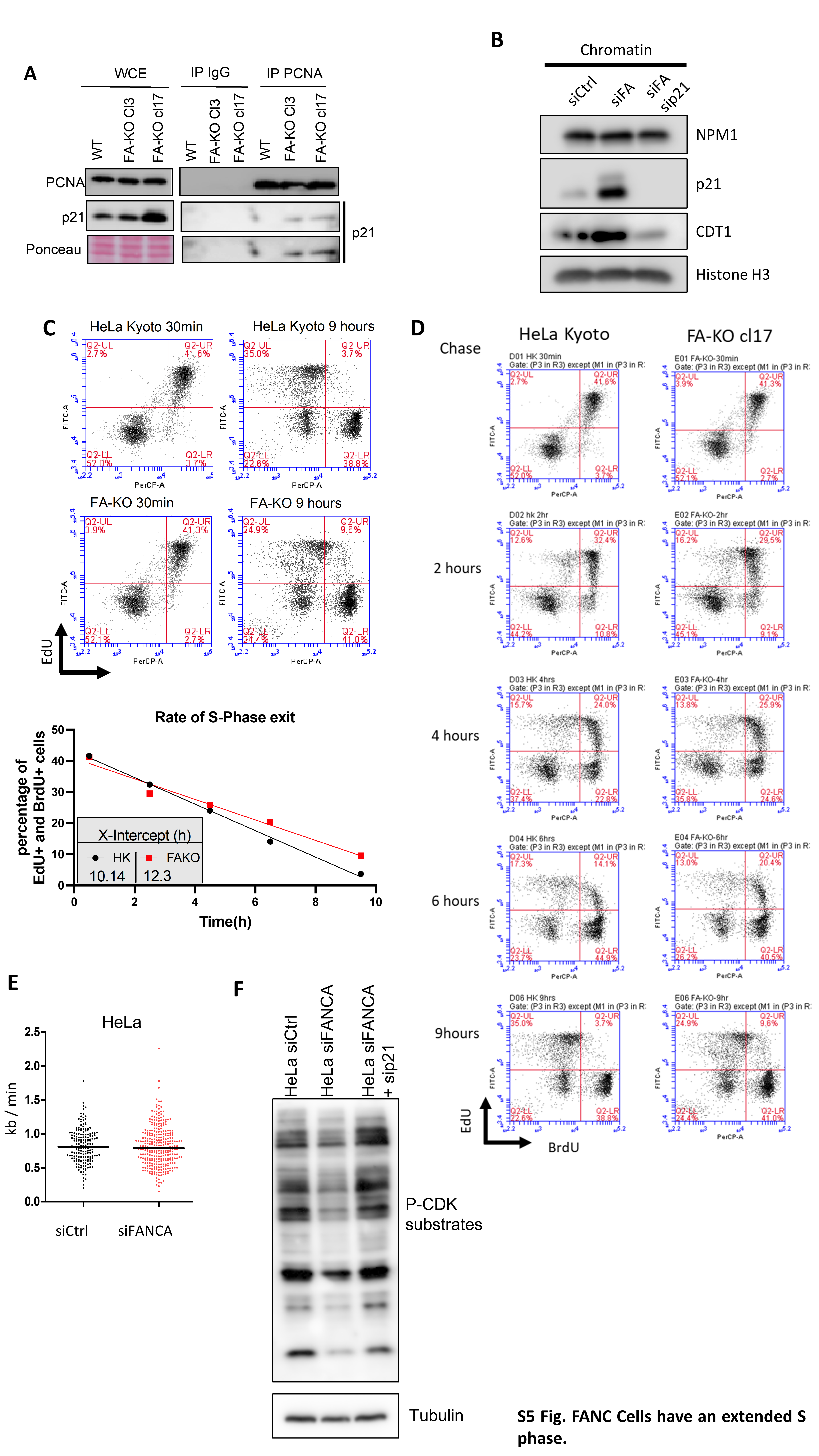

Supplement: S5 Fig — (A) Coimmunoprecipitation of endogenous PCNA with p21. Immunoblots were performed with antibodies against PCNA or p21. (B) Western blot showing the levels of CDT1 and p21 in the chromatin in cells transfected with the indicated siRNAs. (C) Flow cytometry was used to determine the S-phase duration. Asynchronous cells were pulse-labeled for 30 min with 10 μM EdU, washed, and then pulse-labeled again with 100 μM BrdU for 30 min. Samples received the same two pulses but were separated by a thymidine chase period lasting 2 h, 4 h, 6 h or 9 h. Nocodazole was added to prevent progression to the next cycle. Note that the number of double-positive cells (top right quadrant of the middle panel) decreased over time (between 30 min and 9 h). Linear regression of the fraction of EdU+ BrdU+ cells among EdU+ cells over time was used to determine DNA synthesis time as the time when the regression line crossed the x-axis (bottom panel). (D) Flow cytometry was used to determine the S-phase duration. Asynchronous cells were pulse-labeled with 10 μM EdU and then with 100 μM BrdU for 30 min. Samples received the same two pulses but were separated by a thymidine chase period lasting 2 h, 4 h, 6 h, and 9 h. Nocodazole was added to prevent progression to the next cycle. Note that the number of double-positive cells (top right quadrant of the middle panel) decreased over time (between 30 min and 9 h). (E) Analysis of replication fork speed by DNA combing in HeLa cells transfected with siRNA control (siCtrl; n = 174) or siRNA targeting FANCA (siFANCA; n = 314). The graph represents one of two experiments showing comparable results. (F) Western blot showing the levels of phospho-CDK substrates in HeLa cells transfected with siRNA control (siCtrl), targeting FANCA (siFANCA) or targeting both FANCA and p21 (siFANCA/sip21). Tubulin was used as a loading control. (TIF) [file pgen.1011474.s005.TIF]

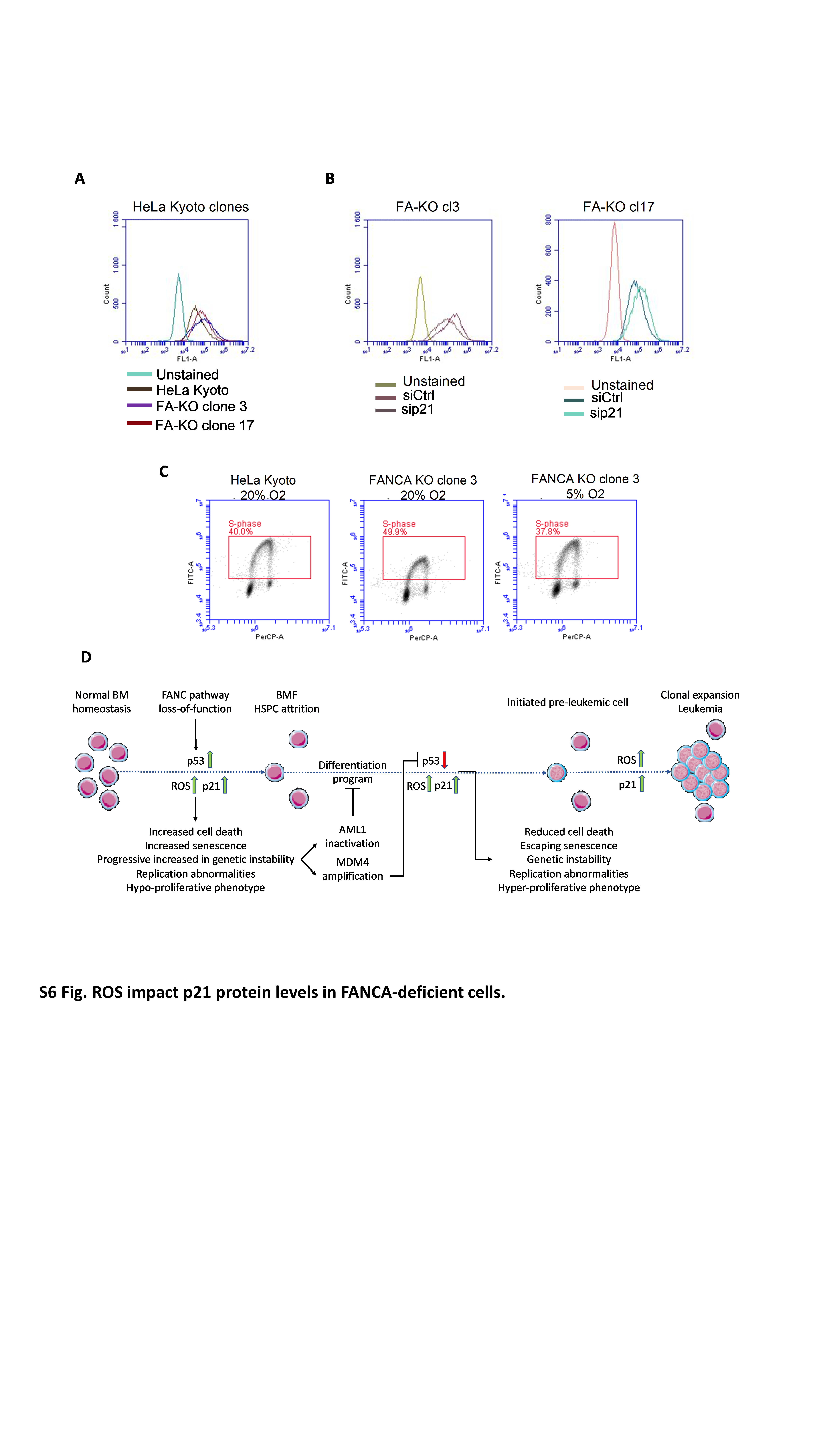

Supplement: S6 Fig — (A) ROS levels in HeLa Kyoto WT and FANCA-KO cell clones 3 and 17 transfected with the indicated siRNA. (B) ROS levels in HeLa Kyoto FANCA-KO cell clones 3 and 17 transfected with the indicated siRNA. Intracellular ROS were measured after incubation with 5 μM CM-H2DCFDA for 15 min. (C) Cell cycle analysis of HeLa Kyoto parental cells and the FANCA knockout clone (clone 3) after 5 days at either 20% or 5% oxygen, as indicated. The cell cycle distribution was revealed by PI/EdU costaining and flow cytometry analysis. (D) Proposed model for the progression from healthy hematopoiesis to leukemia in FA patients. (TIF) [file pgen.1011474.s006.TIF]
